# A data-mining analysis of host solute carrier family proteins in SARS-CoV-2 infection with reference to brain endothelial cells and the blood-brain barrier in COVID-19

**DOI:** 10.3389/fneur.2025.1563040

**Published:** 2025-07-01

**Authors:** Talia Fradkin, Rainald Schmidt-Kastner

**Affiliations:** Schmidt College of Medicine, Florida Atlantic University, Boca Raton, FL, United States

**Keywords:** SARS-CoV-2, COVID-19, blood-brain barrier, brain endothelial cells, solute carrier proteins, amino acid transport, virus-host interactions, protein-protein interactions

## Abstract

**Background:**

The brain vasculature is a key player in neurological manifestations of COVID-19. Infection of brain endothelial cells with SARS-CoV-2 along with circulating cytokines may cause dysfunction of the blood-brain barrier (BBB). Solute carrier transporters (SLCs) in brain endothelial cells regulate substrate transport across the BBB. Here, it was hypothesized that transport functions of SLCs will be impaired by interactions with viral proteins, and subsequently, data-mining studies were performed.

**Methods:**

Virus-host protein-protein interaction data for SARS-CoV-2 infection were retrieved from the BioGRID database, filtered for SLCs, and then annotated for relevant expression in brain endothelial cells using a mouse brain transcriptomics database. Host SLCs expressed in brain endothelial cells were further explored using publicly available databases and information in the literature. Functional Annotation Clustering was performed using DAVID, and Enrichr served for pathway analysis. Substrates were retrieved from NCBI Gene. Links to monogenic disorders were retrieved from Online Mendelian Inheritance in Man™ and screened for disorders of the nervous system. Interactome data for viral proteins of SARS-CoV-2 were retrieved from BioGRID. Reports for host SLCs in viral receptor functions, viral entry mechanisms, and other major roles in the viral cycle were explored in databases (VThunter) and literature. ATP-binding cassette transporters (ABCs) were studied in parallel.

**Results:**

*N* = 80 host SLCs showed relevant expression in brain endothelial cells whereby amino acid transporter stood out. *N* = 24/80 host SLCs were linked to monogenic disorders of the nervous system. *N* = 9/29 SARS-CoV-2 viral proteins had strong links to SLCs and key functions in viral infection (e.g., interferon response). SLCs serving as viral receptors and with closely associated functions were significantly enriched among all known listed viral receptors (chi-square test, *p* = 0.001). Literature searches for host SLCs revealed involvement of a subset of SLCs in infection mechanisms for SARS-CoV-2 and more broadly for other viruses. *N* = 17 host ABCs were found in brain endothelial cells where they may serve as efflux transporters.

**Discussion:**

This hypothesis-generating work proposes a set of *N* = 80 host SLCs expressed in endothelial cells as contributors to BBB impairment after SARS-CoV-2 infection. Theoretically, persistent dysfunction of SLCs at the BBB, in particular insufficient transport of amino acids, could be one of many reasons for cognitive changes in long-COVID. Functions of SLCs in viral entry and associated roles deserve close attention.

## Introduction

The coronavirus, SARS-CoV-2 (Severe Acute Respiratory Syndrome Coronavirus 2), caused a pandemic from early 2020 through mid 2023 and is now considered an endemic virus (1–3). The clinical manifestations of SARS-CoV-2 infection have been named COVID-19 (Coronavirus Disease of 2019), and pulmonary involvement is the leading cause for severe disease and mortality (4, 5). Neurological symptoms and structural damage of the brain (“Neuro-COVID”) were found in a subset of patients acutely affected by COVID-19 (6–10). The mechanisms of brain involvement have been extensively reviewed (9, 11–14). The overall conclusion of these reviews is that viral RNA or proteins can be variably found in the brain, indicating infection of neurons and other cells, but there is limited evidence for replication in neurons or other cells. Little (if any) evidence exists for virus-induced neuronal loss. Neuroinflammation has been also found that does not closely correlate with the detection of viral RNA or protein in the brain, suggesting systemic effects of high levels of cytokines. Ischemic and hypoxic changes are variably found, reflecting systemic effects and local vascular pathology. Functional changes known as Long COVID also appear to affect the brain (15, 16). Thus, SARS-CoV-2 infection may exert functional effects on the brain without leading to overt damage. The nature of these functional effects remains to be defined.

Several lines of evidence converge on the brain vascular system as a key player in COVID-19 (17–26). SARS-CoV-2 may affect endothelial cells through cellular infection (i.e., “endotheliopathy”) or more indirectly through excessive cytokine signaling (or both). Involvement of brain endothelial cells and pericytes may lead to disturbances of the blood brain barrier (BBB) (27–33). Thereby, the invasion of the virus into the brain after the breakdown of the BBB (i.e., hematogenic spread) has attracted most of the attention, while other aspects of the BBB such as controlled substrate influx and efflux are also important.

Solute carrier transporters (SLCs) are a group of membrane transport proteins that carry specific substrates across biological membranes (34–36). SLCs expressed in brain endothelial cells are part of the BBB and selectively transport substrates that are essential for neurons and glial cells (37). Among these are amino acids serving as metabolites, precursors for protein synthesis, and neurotransmitters. The effects of viral infection on SLCs in endothelial cells and BBB could be functional if no cell death occurs in the vasculature. This concept is of particular interest in relation to Long COVID, which can manifest as neurological symptoms, including fatigue and brain fog (15, 16, 38). Several studies have demonstrated BBB changes in Long COVID (14, 29, 39). In addition, studies of various viruses (different from coronavirus) have identified specific SLCs as primary viral receptors or closely associated proteins (40). SLCs for amino acid transport (41, 42) or glucose transport (43) have been identified as viral receptors. Most recently, SLC1A5 has been experimentally shown to participate in SARS-CoV-2 infection (44). Therefore, SLCs could be also involved in the infection process of brain vascular cells. Notably, two amino acid transporters, SLC6A19 and SLC6A20, interact physiologically with ACE2 (Angiotensin Converting Enzyme 2), the key receptor for SARS-CoV-2, in the intestine (45). SLC6A20 has been genetically associated with the clinical course of COVID-19 in multiple GWAS (Genome-wide association studies) (46) and is expressed in brain vasculature (47). While SLCs at the BBB have been predominantly associated with transport into the brain, several ATP-binding cassette transporters (ABCs) in brain endothelial cells move substrates and drugs from the brain across the BBB and into the blood (48). Such efflux could become important if toxins are produced in the brain during infection.

In order to gain a better understanding of the pathophysiology of COVID-19 and to develop drug therapies, major efforts have been undertaken to analyze the interactions between SARS-CoV-2 and host proteins (49). Many viruses can infect the brain whereby viral proteins target host proteins (in neurons or glial cells) to inactivate them or redirect their functions (50). While the focus has been on replication mechanisms, other cellular functions such as substrate transport and protein synthesis are also affected whereby host SLCs could play a role. A large amount of “omics” data collected for SARS-CoV-2 infection has been made publicly available for data-mining (51–53). Proteomics data for virus-host protein interactions in SARS-CoV-2 cellular infection models are available in the public domain through the “SARS-CoV-2 and Coronavirus-Related Interactions” database in BioGRID (Biological General Repository for Interaction Datasets) (54). Combining this resource with other data collections, we carried out a data-mining study and generated a collection of SLCs expressed at the BBB that could be impaired by specific virus-host interactions during SARS-CoV-2 infection in brain endothelial cells. In the first step, information for SLCs as host proteins in SARS-CoV-2 infection was extracted from BioGRID (54). In the second step, expression of these putative virus-interacting SLCs was determined in brain endothelial cells using single cell RNA sequencing data (scRNA seq) as reported by Rosenberg et al. (47) for young mice. The list of virus-interacting host SLCs was further screened in literature and databases to consolidate expression and function in brain endothelial cells and BBB. Subsequently, the list of virus-interacting host SLCs expressed in endothelial cells was screened against various datasets and literature information for infection mechanisms and brain involvement involving seven steps. (1) The DAVID analysis tool was used for Functional Annotation Clustering, resulting in the quantitative classification of groups of SLCs according to their functions. (2) Substrates transported by host SLCs were retrieved from databases. (3) Interactome data were generated for all individual viral proteins of SARS-CoV-2, host SLCs identified, and tested for preferential interaction of SLCs with specific viral proteins. (4) To identify host SLCs critical for brain function, it was assumed that inactivation of host SLCs by viral proteins may have similar effects as disease-causing mutations of the same host proteins, and to this end, host SLCs were annotated for monogenic nervous system disorders using the OMIM™ (Online Mendelian Inheritance in Man) database. (5) Datasets reported for CRISPR (Clustered Regularly Interspaced Short Palindromic Repeats) screening of SARS-CoV-2 infected cell lines were screened for reporting of critical host SLCs. (6) Following a lead from the DAVID analysis, roles of SLCs in viral infection processes, including viral receptor functions, viral entry mechanisms, and other major roles in the viral cycle, were explored in databases and literature, first specifically for SARS-CoV-2, and then more generally for other viruses. (7) Finally, SLCs were compared to ABC transporters expressed in endothelial cells.

## Materials and methods

**Figure 1:**
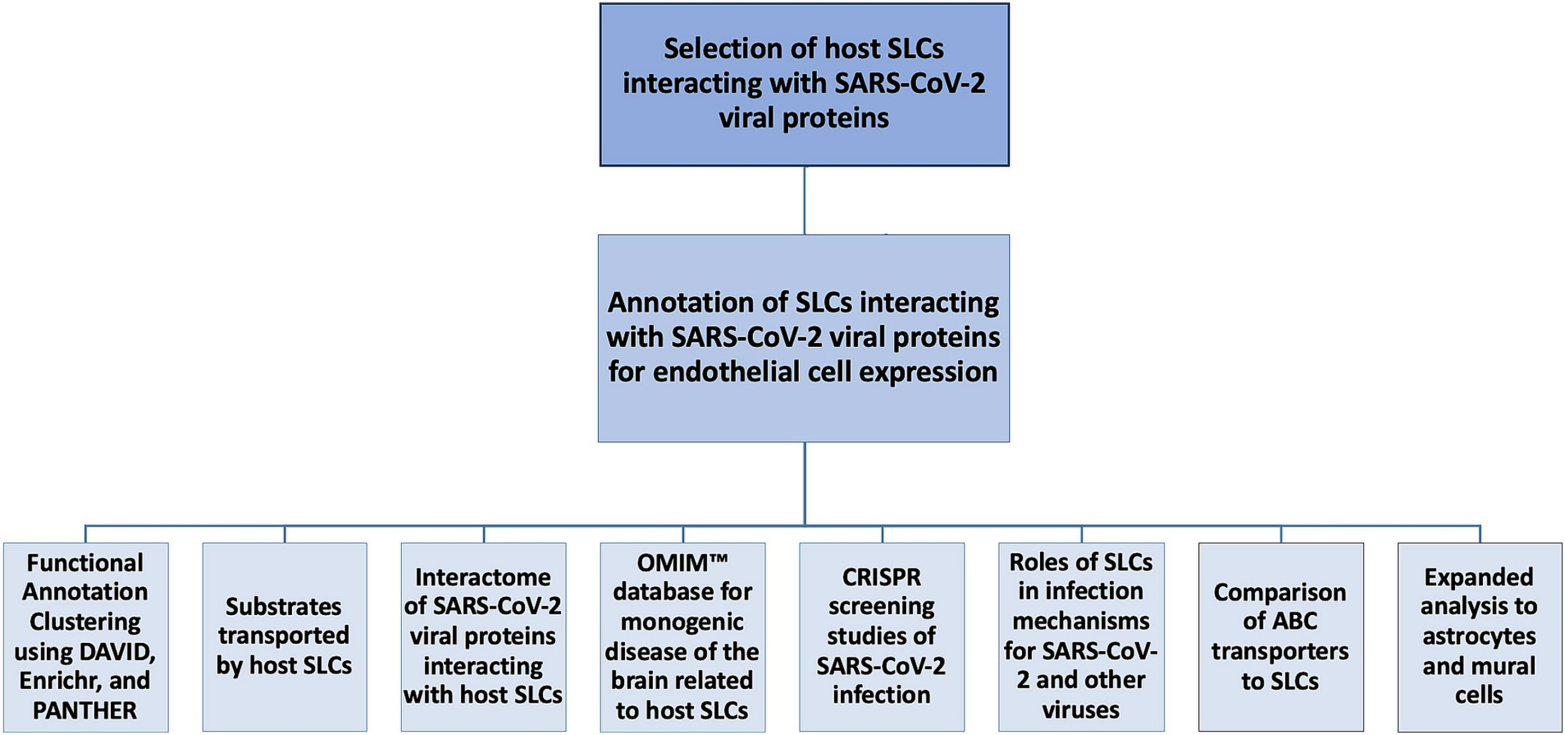
Flow chart of the data-mining process. Solute carrier proteins (SLCs) interacting with Severe acute respiratory syndrome coronavirus 2 (SARS-CoV-2) viral proteins were identified in the virus-host protein–protein interaction (VH-PPI) data. Several analyses were conducted, including a Functional Annotation Clustering analysis using the Database for Annotation, Visualization, and Integrated Discovery (DAVID); substrate classification using the National Center for Biotechnology Information (NCBI) Gene database; viral protein interactome identification using Biological General Repository for Interaction Datasets (BioGRID); diseases of the brain using Online Mendelian Inheritance in Man (OMIM™); Clustered Regularly Interspaced Short Palindromic Repeats (CRISPR) studies; roles of infection based on current literature; a comparison to ATP-binding cassette (ABC) proteins, and an expanded analysis to astrocytes and mural cells.

The workflow of this study has been outlined in Figure 1.

### Selection of host SLCs interacting with SARS-CoV-2 viral proteins

The BioGRID database for proteomics data (54) provides the COVID-19 Coronavirus Curation Project, including the “SARS-CoV-2 and Coronavirus-Related Interactions” database (55). The file called “BIOGRID-PROJECT-covid19_coronavirus_project-LATEST.zip” was downloaded from BioGRID containing the September 1st, 2024 program update. The list was filtered so that “Organism Name Interactor A” and “Organism Name Interactor B” were limited to SARS-CoV-2 and/or *Homo sapiens*; this list of interaction data of SARS-CoV-2 viral proteins with human host proteins will be named virus-host protein–protein interaction (VH-PPI). The list was further filtered by using the first three letters “SLC” of the gene/protein symbols that have been uniformly assigned to denote solute carrier proteins as a large protein family, including “SLCO”; a few scaffold and adaptor proteins were retained. Validity of the gene/protein symbol was verified using the PANTHER (Protein ANalysis THrough Evolutionary Relationships) database (56), which includes the UniProtKG number and symbol. The number of reported listings was recorded for each extracted SLC protein. After removing duplicates, a final list of host SLCs was generated. To estimate possible enrichment within datasets, the total number of known SLCs was taken as *n* = 400 from a key review (34). The number of human proteins interrogated by proteomics techniques and appearing in the combined interactome studies was estimated at 12 k (out of 20 k protein-coding genes). It was assumed that the database of VH-PPI is strongly dominated by infection studies using the original strain and major variants circulating in 2020–21.

### Annotation of SLCs interacting with SARS-CoV-2 viral proteins for endothelial cell expression

Single cell RNA sequencing data (scRNA seq) for cell classes of young mouse brain (including endothelial cells) were downloaded from a database that employed the split-pool ligation based transcriptomics sequencing (47). Data for *n* = 382 SLCs were available in this transcriptomics database. Next, the list of SLCs extracted from VH-PPI was merged with the list of *n* = 382 SLCs in the transcriptomics database and shared genes/proteins determined; human gene/protein symbols will be used during the subsequent reporting. A few host SLC proteins without expression information in the transcriptomics database were removed from the analysis. The selection of SLCs (serving as host proteins) with expression in mouse brain was named “SLC-H.” In the next step, SLCs with relevant expression in brain endothelial cells were determined using the scRNA seq data (column 64 of Table S5, “endothelia”) (47). To this end, a low threshold was set an expression level of *n* = 10 TPMs (Transcripts per Million), and the set of selected genes was named “SLC-H-E.” For comparison, the total number of SLCs obtained with the same threshold for endothelial cell expression (without selection for VH-PPI) was also determined. Literature searches for known roles in brain were carried out to confirm the vascular expression and functional roles in the BBB.

### Functional annotation clustering

The data extracted for SLCs was further analyzed using DAVID (Database for Annotation and Integrated Discovery) (57, 58). This bioinformatics platform provides analysis tools for investigation and annotation of large gene lists for various categories including GO terms and UniprotKB keywords. The list of SLC-H-E was entered using “Official Gene Symbol,” “*Homo sapiens*” as background, and “Gene List” as selectors. The list of genes assigned with DAVID IDs was analyzed using the Functional Annotation Clustering tool, and clusters were provided with an enrichment score. Each cluster then reported a set of preset terms related to a specific functional annotation, such as GO terms with statistical information. Listed terms with a statistically significant enrichment following Bonferroni correction (at *p* < 0.05) were considered for the purpose of this study with a focus on GO terms. This approach allowed for the classification of different SLCs according to substrates, and in addition, uncovered functions of SLCs in virus entry. For a second approach, the Enrichr database was used (59–61). The list of SLC-H-E (*n* = 80) was entered, and the KEGG 2021 Human Pathway option was selected. SLCs were grouped according to functional category, and a Fisher Exact Test with adjusted *p*-value per Benjamini-Hochberg method and odds ratios were generated. As a third approach, the information under PANTHER Protein Class was compiled, and the number of SLCs in different protein classes was determined.

### Substrates transported by selected host SLCs interacting with SARS-CoV-2 viral proteins in brain endothelial cells

Following the detection of several classes of SLCs in the DAVID analysis, a more detailed annotation of individual genes in SLC-H-E for the transported substrates was performed using the summary information provided by NCBI Gene (National Center for Biotechnology Information) (62). The outcome of this analysis was then checked against a specific database named “SLC Tables” at bioparadigms.org (63) that lists detailed information for SLCs, including aliases, transport type and substrates, along with references (mostly dated 2013). Thereby, it must be recognized that substrates remain uncertain or even unknown for a small number of SLCs; these were excluded from the tabulation and graphic presentation. SLCs were grouped by substrates, and expression levels in endothelial cells for each group were compared to “all” other SLCs within SLC-H-E, using unpaired t-test (*p* < 0.05; EXCEL Data Analysis tool).

### Interactome of SARS-CoV-2 viral proteins interacting with host SLCs in brain endothelial cells

While most of the attention has been on the spike protein (S) of SARS-CoV-2, multiple viral proteins play a major role in the infection process. It was asked whether SLCs interacted preferentially with specific viral proteins that in turn have specific functions in controlling the immune response of host cells. BioGRID provided specific PPIs for each of the n = 29 viral proteins of SARS-CoV-2 (E, M, N, nsp1-nsp16, ORF3a, ORF3b, ORF6, ORF7a, ORF7b, ORF8, ORF9b, ORF9c, ORF10, ORF14, S); definitions and functions are found in recent reviews (64, 65). Following the removal of virus-virus interactions, the total number of interacting host proteins was determined for each viral protein, and then matches for the list of SLC-H-E identified. The relative contribution of SLCs to all proteins was calculated for each viral protein and expressed as %. The random chance was estimated as n = 400 SLC proteins in a total of 12 k proteins (measured by proteomics), or 3.3%. Fisher’s exact tests were then carried out for each viral protein to estimate whether SLCs from the SLC-H-E list were randomly found, and viral proteins with significant underrepresentation (*p* < 0.002, corrected for multiple comparisons) excluded. Next, the selected viral proteins linked to SLC-H-E were examined for known roles in virus-mediated interference with host antiviral defenses (including interferon response). To this end, a recent review of the innate immune evasion strategies of SARS-CoV-2 was consulted (65). Textual information for *n* = 23 SARS-CoV-2 viral proteins (provided in Table 1 of the publication) was extracted, including the classification of “activities” of viral proteins. After combining and sorting, the three top “activities” were selected (“Blocks recognition by host sensors,” “Minimizes or masks inflammatory RNA,” “Blocks IFN signaling”) and then tabulated for the viral proteins linked to SLC-H-E.

### Analysis of OMIM™ database for monogenic disease of the brain related to host SLCs interacting with SARS-CoV-2 viral proteins

To further prioritize interacting SLCs in relation to brain disease mechanisms, it was hypothesized that interactions with viral proteins may have similar inactivating effects on host SLCs as known mutations. Accordingly, SLC-H-E were screened for known mutations causing (monogenic) brain disorders using the OMIM™ database (66). Some genes affecting the peripheral nervous system and sensory organs were included. Most genes causing nervous system disorders were readily recognized by their “official name.” For some syndromes more detailed information for nervous system symptoms was retrieved from the “Clinical Synopsis” page associated with the entry for the disease. The analysis was then extended to all genes from SLC-H. Literature searches were used to supplement the analysis.

### SLCs listed in CRISPR screening studies of SARS-CoV-2 infection

CRISPR techniques using array formats allow to knock-down host genes which can result in the identification of novel pro- or antiviral functions of host proteins (67). Accordingly, the literature reporting data for CRISPR approaches to SARS-CoV-2 infection was surveyed for reports of SLCs. Two studies reviewing previous results were analyzed first (67, 68) and then individual articles were scanned. The data were highly heterogenous across different publications due to different cell types used, variation of the CRISPR tools, and selection or presentation of significant hits, and therefore only few SLCs will be reported here.

### Roles of SLCs in infection mechanisms for SARS-CoV-2 and other viruses

Based on the annotation of SLCs for virus entry detected by the DAVID bioinformatics analysis (reported below under Results), a broader search was carried out for links between selected SLCs and viruses. First, a collection of viral receptors in the “VThunter” database (40, 69) was examined for SLCs broadly involved in viral infections. Then, each SLC-H-E was screened using PubMed abstract searches that combined the search term “virus” with the gene symbol for each SLC. Known roles in viral receptor functions, viral entry mechanisms, and other major roles in the viral cycle were identified. The initial search was directed at experimental studies of SARS-CoV-2, and it was then extended more broadly to other viruses.

### Analysis of ABC transporters expressed in brain endothelial cells and interacting with SARS-CoV-2 proteins

Several ABC (ATP-binding cassette) transporters in brain endothelial cells move substrates and drugs from the brain over the BBB and into the blood (48). Theoretically, such efflux transporters may become relevant when viral infections lead to a build-up of toxic levels of small molecules within the brain. Accordingly, the list of VH-PPI (as generated before) was screened for host ABC transporters that interact with viral proteins. To this end, the letters “ABC” of the gene/protein symbol shared by this protein family were used for selection. PANTHER (56) was used to verify the gene/protein symbols. Subsequently, expression of selected ABC transporters in mouse brain endothelial cells was determined using the process described above for SLCs, including a cut-off at 10 TPM. A published data collection for *n* = 50 ABCs expressed at the BBB was retrieved and used to confirm the selections based on the transcriptomics data (48). Two-sample t-tests assuming equal variances were conducted for the number of entries of ABCs (using a cut-off at 10 TPM) compared to those for SLC-H-E; and for the expression levels in endothelial cells in both sets.

### Expanded analysis of datasets including astrocytes and mural cells

The BBB function also involves astrocytes and mural cells (e.g., vascular leptomeningeal cells, VMLC), and SLCs expressed by these cell types may also be affected by viral proteins. Thereby, it is of interest to determine which SLCs defined for brain endothelial cells (SLC-H-E) are shared with these other cells and which ones are specific. To this end, datasets for astrocytes (column 70, “Astro GFAP” of table S5 (47)) and mural cells (column 66, “VLMC Slc6a13”) were downloaded, selected for presence on the SLC-H list, and categorized at an expression level of *n* = 10 TPMSs, using the same approach as for brain endothelial cells. The datasets for selected SLCs for the three cell types were then overlapped with shared genes determined and visualized in a Venn diagram. In addition, the viral proteins interacting with different host SLCs from this analysis were determined (as already described above for the analysis of endothelial cells) and tabulated for each cell type. ANOVA was used to examine the difference in the number of interactions between viral proteins and SLCs across the three cell types. Viral proteins interacting with SLCs in all three cell types were defined as targets of high interest.

## Results

### Host SLCs interacting with SARS-CoV-2 viral proteins

The file “BIOGRID-PROJECT-covid19_coronavirus_project-LATEST.zip” was downloaded from BioGRID and filtered for SARS-CoV-2 and human studies; the selection of SARS-CoV-2 viral proteins interacting with human host proteins led to *n* = 39,854 total entries and *n* = 6,993 singular host proteins. *N* = 134 SLCs were found among the host proteins by filtering with the first three letters of the gene/protein symbol. The number of listings (or “entries”) per SLC in the VH-PPI was determined, which may serve as a proxy for the relative importance of different SLCs. *N* = 45 SLCs had 10 or more interactions with the viral proteins listed. It is estimated that with about *n* = 400 SLCs in total described in the literature (34), one third of all SLCs have been listed as host proteins for SARS-CoV-2 (in VH-PPI). Assuming that 12 k “measurable” proteins were captured by the proteomics studies in the database and that host proteins in the VH-PPI (6.9 k) are about half of these, no relative enrichment for SLCs could be found among host proteins. To obtain independent confirmation for the validity of the SLC nomenclature, the gene list selected from BioGRID was entered into PANTHER for verification, including UniProt KB numbers and symbols; all selections made in BioGRID were matched in PANTHER (Supplementary material 1).

### Host SLCs interacting with SARS-CoV-2 viral proteins annotated for endothelial cell expression

Subsequently, the database of Rosenberg et al. (47) for young mouse brain reporting scRNA seq data for *n* = 382 SLC genes was used for annotation. *N* = 134/382 genes (or 35%) were on the list of SLCs generated from the VH-PPI. Four genes (SLC18A3, SLC25A52, SLC25A6, SLCO6A1) were not in the mouse transcriptomics study and removed. The remainder of *n* = 130 genes/proteins will be named “SLC-H.” Next, the host SLCs expressed in mouse brain endothelial cells were determined. It was decided to limit further analyses to an expression level of *n* = 10 TPMs or higher in brain endothelial cells which resulted in *n* = 80 genes/proteins named “SLC-H-E.” The dataset SLC-H-E (*n* = 80) was analyzed in a number of subsequent studies; for some analyses, the additional genes in SLC-H were also considered as specified below. Table 1 lists the findings, with SLC-H-E marked in bold.

**Table 1:** Solute carrier proteins in virus-host protein–protein interactions.

**SLC1A1**	**SLC4A1AP**	**SLC7A7**	**SLC12A7**	**SLC20A1**	SLC25A19	**SLC26A11**	**SLC31A1**	SLC37A4	**SLC44A1**
**SLC1A3**	SLC5A2	**SLC7A11**	SLC12A9	**SLC20A2**	**SLC25A20**	SLC27A2	**SLC33A1**	**SLC38A1**	**SLC44A2**
**SLC1A4**	**SLC5A3**	**SLC9A1**	**SLC15A4**	SLC22A3	SLC25A21	SLC27A3	**SLC35A1**	**SLC38A2**	**SLC44A5**
**SLC1A5**	**SLC5A6**	SLC9A3R1	**SLC16A1**	**SLC22A5**	SLC25A22	**SLC27A4**	SLC35A2	**SLC38A9**	SLC45A1
**SLC2A1**	**SLC6A6**	**SLC9A3R2**	SLC16A3	**SLC23A2**	**SLC25A23**	**SLC29A1**	SLC35B1	**SLC38A10**	SLC45A4
SLC2A3	SLC6A8	**SLC9A6**	**SLC16A4**	SLC25A1	**SLC25A24**	SLC29A2	SLC35B2	**SLC39A6**	SLC46A1
SLC2A6	SLC6A9	SLC9A7	SLC16A5	SLC25A2	**SLC25A3**	**SLC29A3**	SLC35C2	SLC39A7	SLC47A1
**SLC2A13**	**SLC6A15**	**SLC9A8**	**SLC16A6**	SLC25A10	**SLC25A4**	**SLC30A1**	**SLC35D1**	**SLC39A10**	SLC47A2
**SLC3A2**	**SLC6A17**	SLC9B2	**SLC16A10**	SLC25A11	SLC25A5	**SLC30A4**	**SLC35E1**	**SLC39A11**	SLC51B
**SLC4A2**	**SLC7A1**	SLC10A1	**SLC17A5**	**SLC25A13**	**SLC25A46**	**SLC30A5**	**SLC35F2**	SLC39A14	SLC52A2
**SLC4A4**	**SLC7A2**	**SLC12A2**	**SLC18B1**	SLC25A15	**SLC26A2**	**SLC30A6**	**SLC35F5**	SLC41A2	**SLCO3A1**
**SLC4A7**	**SLC7A5**	**SLC12A4**	**SLC19A1**	SLC25A16	SLC26A4	**SLC30A7**	SLC35F6	SLC41A3	SLCO4A1
SLC4A10	**SLC7A6**	**SLC12A6**	SLC19A2	**SLC25A17**	**SLC26A6**	**SLC30A9**	SLC35G2	SLC43A1	SLCO4C1

Host solute carrier proteins (in total *n* = 130; SLC-H) found in the virus-host protein–protein interaction (VH-PPI) data are displayed in numerical order. The subset of host solute carrier proteins found at relevant levels in the transcriptome of mouse brain endothelial cells (*n* = 80; SLC-H-E) is indicated in bold.

**Figure 2:**
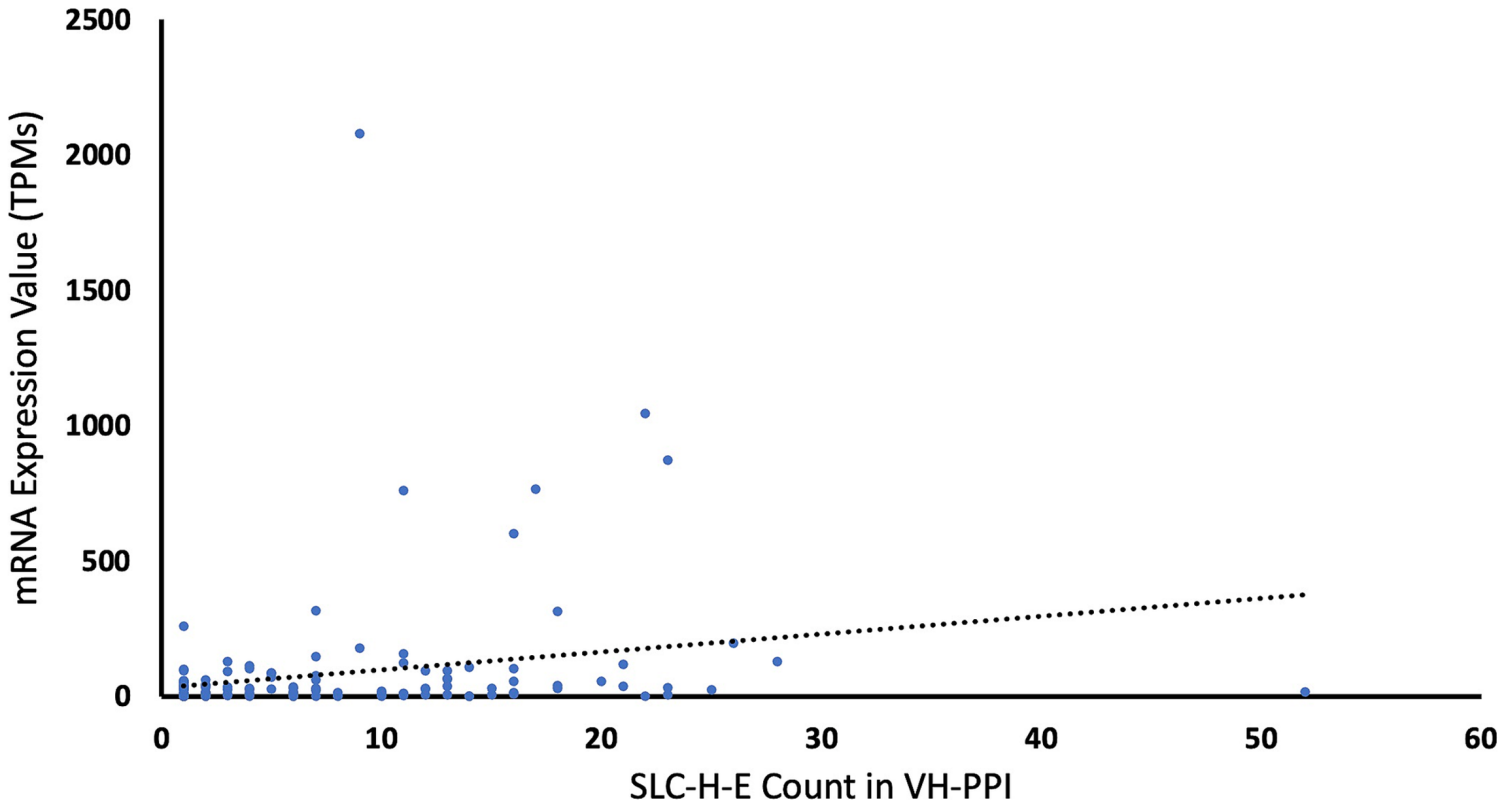
Counts of SLCs in virus-host protein–protein interaction data versus mRNA expression values. The chart displays the counts of each SLC-H-E in the virus-host protein–protein interaction data plotted against its brain endothelial expression value. The dotted line for linear fit indicates low correlation (*r* = 0.2186).

Two tests were carried out to look for bias that could arise from the selection based on expression levels. First, proteins with high expression levels may have more chances to interact with other proteins and, hence, may show more entries in VH-PPI. To probe for such bias, the mRNA expression levels in endothelial cells (assuming proportional protein expression) were plotted against the counts for each SLC-H-E in the VH-PPI, and a very low correlation was found (*r* = 0.2186; Figure 2). Second, the expression levels of SLC-H-E were compared to those of SLCs not in the VH-PPI using the same threshold for endothelial cell expression (at 10 TPM); no difference in expression between these two gene sets was found (t-test, *p* = 0.58).

A literature survey showed that several SLC-H-E genes/proteins are already known to have brain endothelial cell expression and major BBB functions or they relate to vascular mechanisms relevant to COVID-19. For example, SLC5A6 is involved in pantothenic acid uptake by brain endothelial cells (70). SLC7A5/LAT1 (large neutral amino acids transporter small subunit) (1) is involved in L-leucine transport at the BBB and also serves as a major drug transporter, including transport of L-DOPA (71). SLC9A1 is key to regulating intracellular pH in endothelial cells, and subsequently, for the integrity of the BBB (72). SLC44A1 and SLC44A2 are implied in choline transport through the BBB (73). GWAS have suggested a role for SLC44A2 in regulating thrombosis, which involves mitochondria in platelets (74); this is of particular interest as formation of microthrombosis in cerebral vessels is considered a major cause of brain pathology in COVID-19. In addition, expression at the protein level could be confirmed for *n* = 34/80 SLC-H-E in a proteomics study of adult mouse brain capillaries (75).

### Functional annotation clustering identifies classes of SLCs

The Functional Annotation Clustering tool provided multiple hits for SLC-H-E and terms of interest with significant enrichment after Bonferroni correction (*p* < 0.05) as shown in Table 2. In the category of GO terms, significant results were related to amino acid transmembrane transporters, zinc ion transport, and regulation of intracellular pH. *N* = 8 SLCs were classified under the GO term “neurotransmitter transport.” INTERPRO annotation was found for choline transport. The annotation term “Host cell receptor for virus entry” (“UP_KW_MOLECULAR FUNCTION,” derived from UniProt) listed SLC1A5, SLC3A2 and SLC20A2 (Bonferroni, *p* = 0.005). The related GO term “virus receptor activity” listed SLC1A5, SLC3A2, SLC7A1 and SLC20A2, for which basic enrichment analysis was listed as significant at *p* = 0.004 but did not pass Bonferroni correction (*p* = 0.6). The results of the Enrichr analysis are provided in Supplementary material 2. Notable findings by Enrichr (59–61) were “protein digestion and absorption,” “choline metabolism in cancer,” and “glutamatergic synapse,” which corresponds to annotations using DAVID. In addition, the qualitative information for PANTHER (56) protein classes was compiled (Supplementary material 1): secondary carrier transporter (*n* = 29); transporter (*n* = 25), primary active transporter (*n* = 7); amino acid transporter (*n* = 6), and mitochondrial carrier proteins (*n* = 3). While this classification is different from the ones provided using GO terms in DAVID, it introduces the new aspect of secondary transport, which depends on gradients produced by energy-dependent ion pumps.

**Table 2:** List of results of Functional Annotation Clustering.

Terms	Count	%	*p*-value	Fold enrichment	Bonferroni
GO:0003333 ~ amino acid transmembrane transport	10	12.66	8.36E-16	90.36	5.05E-13
GO:0015179 ~ L-amino acid transmembrane transporter activity	8	10.13	9.03E-13	98.99	2.07E-10
GO:0005283 ~ amino acid: sodium symporter activity	3	3.80	1.56E-04	148.49	0.035059
GO:0032328 ~ alanine transport	5	6.33	7.43E-09	180.71	4.23E-06
GO:0061459 ~ L-arginine transmembrane transporter activity	5	6.33	1.13E-07	103.12	2.59E-05
GO:1903826 ~ L-arginine transmembrane transport	5	6.33	2.83E-07	84.33	1.61E-04
GO:0015173 ~ aromatic amino acid transmembrane transporter activity	3	3.80	9.37E-05	185.62	0.021239
GO:0140009 ~ L-aspartate import across plasma membrane	4	5.06	1.13E-06	168.67	6.44E-04
GO:0015183 ~ L-aspartate transmembrane transporter activity	4	5.06	5.04E-06	109.99	0.001153
GO:0015174 ~ basic amino acid transmembrane transporter activity	4	5.06	3.37E-06	123.74	7.71E-04
GO:0015813 ~ L-glutamate transmembrane transport	5	6.33	1.22E-06	60.24	6.93E-04
GO:0006868 ~ glutamine transport	5	6.33	2.66E-08	140.56	1.51E-05
GO:0015186 ~ L-glutamine transmembrane transporter activity	5	6.33	2.90E-08	137.49	6.65E-06
GO:0015804 ~ neutral amino acid transport	10	12.66	6.15E-17	115.00	6.32E-14
GO:0015823 ~ phenylalanine transport	3	3.80	8.97E-05	189.75	0.049767
GO:0015828 ~ tyrosine transport	3	3.80	4.50E-05	253.00	0.025263
KW-0029 ~ Amino-acid transport	17	21.52	7.99E-23	45.37	2.08E-21
GO:0071577 ~ zinc ion transmembrane transport	9	11.39	2.58E-14	94.88	1.47E-11
GO:0005385 ~ zinc ion transmembrane transporter activity	9	11.39	6.50E-14	85.67	1.49E-11
GO:0071578 ~ zinc ion import across plasma membrane	4	5.06	6.72E-06	101.20	0.003817
GO:0006829 ~ zinc ion transport	4	5.06	6.72E-06	101.20	0.003817
GO:1904257 ~ zinc ion import into Golgi lumen	3	3.80	4.50E-05	253.00	0.025263
GO:0006882 ~ intracellular zinc ion homeostasis	7	8.86	4.29E-09	50.60	2.44E-06
IPR007603: Choline_transptr-like	3	3.80	1.40E-04	156.51	0.016018
GO:0008519 ~ ammonium channel activity	4	5.06	1.31E-05	82.50	0.002991
GO:0140157 ~ ammonium import across plasma membrane	3	3.80	8.97E-05	189.75	0.049767
GO:0015701 ~ bicarbonate transport	7	8.86	6.11E-09	47.86	3.48E-06
GO:1902476 ~ chloride transmembrane transport	11	13.92	1.40E-11	25.53	7.95E-09
GO:0055064 ~ chloride ion homeostasis	4	5.06	9.21E-06	92.00	0.00523
GO:0008509 ~ monoatomic anion transmembrane transporter activity	4	5.06	7.18E-06	98.99	0.001642
GO:0006811 ~ monoatomic ion transport	16	20.25	1.42E-18	31.87	8.06E-16
GO:0071805 ~ potassium ion transmembrane transport	7	8.86	1.72E-05	12.74	0.009758
GO:0055075 ~ potassium ion homeostasis	4	5.06	6.21E-05	50.60	0.034705
GO:0035725 ~ sodium ion transmembrane transport	13	16.46	5.73E-14	26.52	3.26E-11
GO:0098719 ~ sodium ion import across plasma membrane	5	6.33	2.55E-06	50.60	0.001448
GO:0051453 ~ regulation of intracellular pH	7	8.86	1.28E-09	61.07	7.28E-07
GO:0015718 ~ monocarboxylic acid transport	4	5.06	3.73E-05	59.53	0.021023
GO:0008028 ~ monocarboxylic acid transmembrane transporter activity	4	5.06	4.77E-05	55.00	0.010862
GO:0015866 ~ ADP transport	4	5.06	4.72E-06	112.44	0.002681
GO:0000295 ~ adenine nucleotide transmembrane transporter activity	3	3.80	9.37E-05	185.62	0.021239
GO:1990544 ~ mitochondrial ATP transmembrane transport	4	5.06	4.72E-06	112.44	0.002681
GO:0006839 ~ mitochondrial transport	4	5.06	2.02E-05	72.29	0.011404
GO:0006836 ~ neurotransmitter transport	8	10.13	2.52E-10	48.19	1.43E-07
GO:0031902 ~ late endosome membrane	6	7.59	2.47E-04	10.58	0.033093
KW-1183 ~ Host cell receptor for virus entry	3	3.80	7.45E-04	61.92	0.005202

The table displays the results for host SLCs expressed in endothelial cells (SLC-H-E) obtained by Functional Annotation Clustering analysis using the DAVID (Database for Annotation, Visualization, and Integrated Discovery) tool. “Terms” are mainly from GO (Gene Ontology) categories, whereby counts, % and *p*-value are provided for each category, along with fold enrichment and the *p*-value for Bonferroni test (*p* < 0.05).

### Important substrates are transported by selected host SLCs interacting with SARS-CoV-2 viral proteins in brain endothelial cells

**Figure 3:**
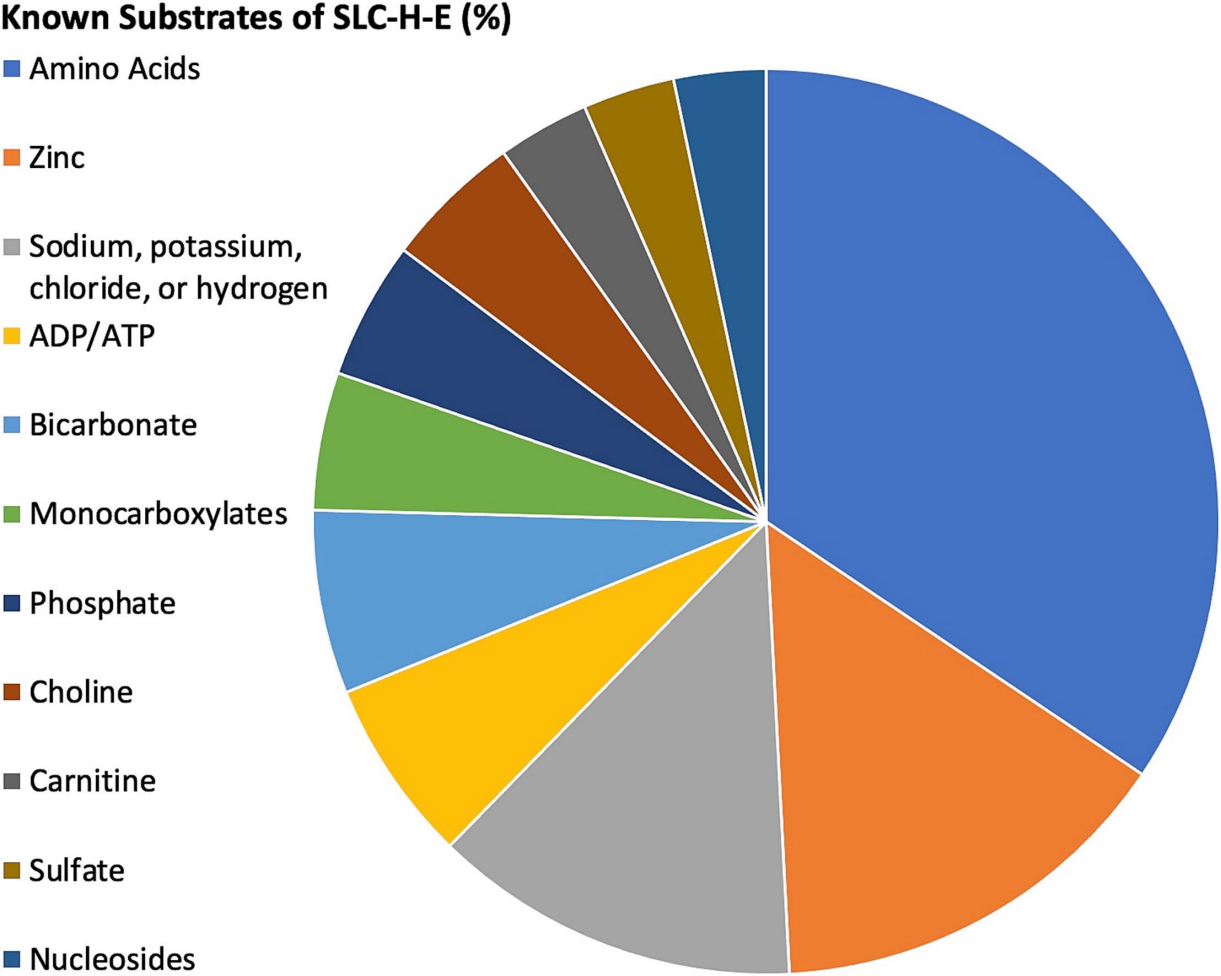
Relative distribution of substrate classes among selected SLCs. The pie chart demonstrates the relative contribution (in percentage) for classes of substrates with at least two members as identified by NCBI Gene for SLC-H-E. SLCs with unknown substrates were excluded. The key to the left shows the color coding for substrate classes. Distribution from highest to lowest were amino acids, zinc, sodium/potassium/chloride/hydrogen, ADP/ATP, bicarbonate, monocarboxylates, phosphate, choline, carnitine, sulfate, and nucleosides. For a complete list, see Table 3.

In the following, the working hypothesis is that the interaction of a viral protein will negatively affect a related host SLC-H-E by reducing its availability for physiological protein interactions in the infected brain endothelial cells. Information for the substrates transported by *n* = 73/80 SLC-H-E was found in the summary information provided in NCBI Gene. Several classes of SLCs emerged, whereby *n* = 21/80 were determined to transport amino acids, *n* = 3/80 choline, and *n* = 9/80 zinc (Table 3; Figure 3). The substrates listed in the specific database named “SLC Tables” (at bioparadigms.org) largely coincided with the information provided under NCBI Gene, and inspection of the annotations identified by DAVID bioinformatics also indicated strong overlap. When comparing the expression levels in endothelial cells, the *n* = 21 SLCs grouped to amino acid transport had significantly higher expression values than all other SLCs (*n* = 59) within the SLC-H-E set (unpaired t-test, *p* = 0.006). This was not observed for SLCs related to zinc transport (*p* = 0.9). An additional analysis was carried out for the five SLC-H-E not annotated with a substrate in NCBI Gene (setting aside SLC4A1AP and SLC9A3R2). Using abstract searches in PubMed, four new annotations were found; SLC18B1 as a polyamine transporter (76); SLC25A46 as a mitochondrial carrier protein (77); SLC35E1 as a regulator of zinc concentration (78); and SLC35F5 as nucleotide sugar transporter (79). Following this, only SLC35F2 remained as an orphan transporter in the SLC-H-E analysis. Interestingly, a detailed study of SLC35F2 in the BBB has appeared (80).

**Table 3:** List of known substrates of host SLCs expressed in endothelial cells.

Substrates	Count	SLC-H-E
Amino Acids	21	SLC1A1, SLC1A3, SLC1A4, SLC1A5, SLC3A2, SLC6A6, SLC6A15, SLC6A17, SLC7A1, SLC7A2, SLC7A5, SLC7A6, SLC7A7, SLC7A11, SLC15A4, SLC16A10, SLC25A13, SLC38A1, SLC38A2, SLC38A9, SLC38A10
Zinc	9	SLC30A1, SLC30A4, SLC30A5, SLC30A6, SLC30A7, SLC30A9, SLC39A6, SLC39A10, SLC39A11
Sodium, potassium, chloride, or hydrogen	8	SLC9A1, SLC9A6, SLC9A8, SLC12A2, SLC12A4, SLC12A6, SLC12A7, SLCO3A1
ADP/ATP	4	SLC25A4, SLC25A17, SLC25A23, SLC25A24
Bicarbonate	4	SLC4A2, SLC4A4, SLC4A7, SLC26A6
Monocarboxylates	3	SLC16A1, SLC16A4, SLC16A6
Phosphate	3	SLC20A1, SLC20A2, SLC25A3
Choline	3	SLC44A1, SLC44A2, SLC44A5
Carnitine	2	SLC22A5, SLC25A20
Sulfate	2	SLC26A2, SLC26A11
Nucleosides	2	SLC29A1, SLC29A3
Glucose	1	SLC2A1
Myo-inositol	1	SLC2A13
Myo-inositol, glucose	1	SLC5A3
Biotin	1	SLC5A6
Sialic acid	1	SLC17A5
Folate	1	SLC19A1
Ascorbic Acid	1	SLC23A2
Fatty acids	1	SLC27A4
Copper	1	SLC31A1
Acetyl-coenzyme A	1	SLC33A1
CMP-sialic acid	1	SLC35A1
UDP-glucuronic acid, UDP-N-acetylgalactosamineT	1	SLC35D1

The table provides the specific substrates of SLC-H-E as determined by NCBI Gene.

### SARS-CoV-2 viral proteins interact with host SLCs in brain endothelial cells

Subsequently, it was asked whether SLC-H-E interact preferentially with a specific set of viral proteins. PPI information for *n* = 29 SARS-CoV-2 viral proteins was downloaded from BioGRID.

**Figure 4:**
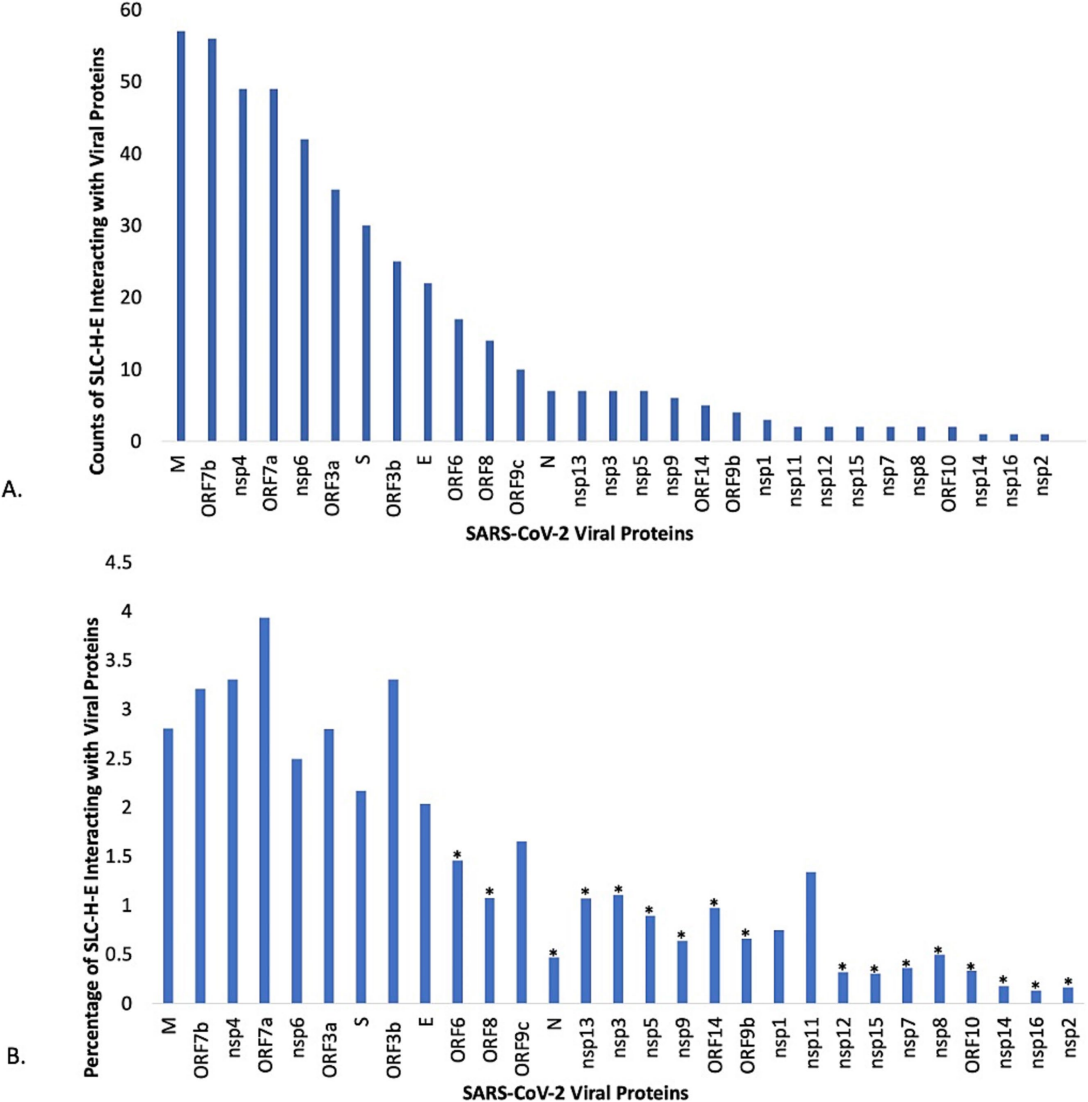
Analysis of individual viral proteins of SARS-CoV-2 for interactions with selected SLCs. **(A)** The plot displays the counts for host SLCs expressed in endothelial cells (SLC-H-E) after removing duplicates, as found in the interactome for each individual SARS-CoV-2 viral protein. **(B)** The plot displays the relative contribution of host SLCs expressed in endothelial cells (SLC-H-E) compared to the total count of host proteins in VH-PPI for each SARS-CoV-2 viral protein. Fisher’s exact tests were carried out for over- or underrepresentation of SLCs relative to the total count of host proteins interacting with each viral protein (* *p* < 0.002, corrected for multiple comparisons).

Following removal of duplicates, the number of host proteins and number of SLC genes from SLC-H-E was determined for each viral protein. Subsequently, the relative contribution of SLC-H-E was calculated and probed using Fisher’s exact test for each viral protein. The distribution according to counts of SLCs from SLC-H-E interacting with each viral protein is shown in Figure 4A, and the relative contribution of SLCs (in %) in Figure 4B. *N* = 17 viral proteins were excluded due to significant underrepresentation of SLCs; none were enriched. *N* = 12 viral proteins linked to SLCs remained for further consideration. Information for *n* = 23 SARS-CoV-2 viral proteins was extracted from a current review including the classification of “activities” of these viral proteins (65). Information for *n* = 9/12 viral proteins linked to SLCs was available for which the three top activities (“Blocks recognition by host sensors,” “Minimizes or masks inflammatory RNA,” “Blocks IFN signaling”) were assigned as listed in Table 4. A list of the SLCs (including their substrates) interacting with these specific viral proteins is provided in Supplementary material 3. Viral proteins blocking the interferon response stood out (*n* = 6/9).

**Table 4:** Functions of selected SARS-CoV-2 viral proteins interacting with host SLCs expressed in endothelial cells.

Viral protein	Blocks recognition by host sensors	Minimizes or masks inflammatory RNA	Blocks IFN signaling
M	X		
ORF7b	X		X
nsp4		X	
ORF7a	X		X
nsp6	X	X	X
ORF3a			X
S			X
ORF3b	X		
nsp1	X		X

The table lists nine SARS-CoV-2 viral proteins linked to host SLCs expressed in endothelial cells that matched categories “Blocks recognition by host sensors,” “Minimizes or masks inflammatory RNA,” and “Blocks IFN signaling,” obtained from a recent review (61) listing *n* = 23 viral proteins related to the innate immune evasion strategies of SARS-CoV-2. More details are found in Supplementary File 3.

### A subset of host SLCs interacting with SARS-CoV-2 viral proteins is linked to monogenic diseases of the brain

The assumption was made that interactions with viral proteins may have similar inactivating effects on SLCs as known genomic mutations in the nervous system, and that this allows for classifying specific SLCs as particularly interesting in the context of functional disturbances. Accordingly, sets of SLCs were annotated for known mutations causing nervous system disorders using the OMIM™ database based on the name of the disorder or information under “clinical synopsis.” *N* = 33/80 SLC-H-E genes were listed for any disorder, and *n* = 24/80 (or 30%) were classified as nervous system disorders. Endothelial cell expression may contribute to the disease process for these genes while other cells in the brain may also be involved (as discussed below under “Multiple host SLCs are shared between endothelial cells, astrocytes, and mural cells”). Therefore, the search was expanded to the remainder of genes in SLC-H, and *n* = 18 SLCs were also classified as linked to nervous system disorders. Taken together, *n* = 42 SLCs among the *n* = 130 in SLC-H (32%) were related to disorders of the nervous system (mostly affecting the brain) as shown in Supplementary material 4.

### CRISPR screening studies of SARS-CoV-2 infection lead to host SLCs

A recent review for CRISPR studies of viral infections was consulted (67) and the genes of interest listed for experimental studies for SARS-CoV-2 retrieved. Among *n* = 180 genes listed, three SLCs were found, one of which was in SLC-H-E (SLC7A11) and another only in SLC-H (SLC35B2). Another report combined their CRISPR based data with findings from the literature (68) and reported *n* = 22 SLC genes, whereby *n* = 5 of these were in SLC-H-E.

Screening of individual studies using CRISPR-based approaches in cells with SARS-CoV-2 infection led to SLC35B2 (81); SLC35F4 (82); SLC5A1, SLC6A14, SLC6A19 and SLC35B2 (83); SLC1A5 and SLC31A1 (84); and SLC1A12, SLC12A9, SLC62A1 and SLC63A1 (85). Taken together, converging evidence from SLC-H and CRISPR studies points to SLC35B2, which has been also reported for other viruses (86).

### SLCs play a role during infection with SARS-CoV-2 and other viruses

Links between SLCs and mechanisms of infection, including viral receptor functions, viral entry mechanisms, and other major roles in the viral cycle, were examined, first for SARS-CoV-2 and then more broadly for other viruses. The database VThunter (40) contained *n* = 108 genes/proteins as viral receptors, out of which *n* = 7 were SLCs (SLC10A1, SLC1A5, SLC20A1, SLC20A2, SLC52A1, SLC52A2, SLC7A1). With *n* = 400 SLCs randomly expected among a total of 20 k genes in the genome, SLCs were estimated as enriched among viral receptors in this database (Chi-square test, GraphPad, two-tailed, *p* = 0.001). When tested against an estimated total of 12 k proteins measured, marginal significance was found (Chi-square test, one-tailed, *p* = 0.04). Four of these SLCs listed in VThunter were found on the SLC-H-E list (SLC1A5, SLC20A1, SLC20A2, SLC7A1) and two on list of SLC-H only (SLC10A1, SLC52A2). As reported above, the analysis using the DAVID bioinformatics tool indicated a significant enrichment for “Host cell receptor for virus entry” among the SLC-H-E selection whereby SLC1A5, SLC20A2, and SLC3A2 were listed; weak evidence for SLC7A1 was found.

In the following section, supportive information for links of SLC-H-E to SARS-CoV-2 infections, specifically, and to viral infections (including other viruses), more generally, will be reviewed. Thereby, genes listed in VThunter and/or obtained from DAVID analysis were given high priority, along with SLCs reported in experimental studies of SARS-CoV-2. SLC1A5 is a neutral amino acid transporter listed in VThunter and detected by DAVID analysis. Experimental studies for SARS-CoV-2 uncovered a role for SLC1A5 as a viral entry modulator of ACE2 (44). SLC1A5 was also listed for protein interactions with the SARS-CoV-2 Spike protein (87). SLC1A5 serves as receptor for the RD-114 virus, a feline endogenous retrovirus (42, 88). SLC3A2 transports amino acids and regulates intracellular calcium that was detected by DAVID analysis. An experimental study in SARS-CoV-2 infected cells searched for host proteins by cell surface proximity ligation techniques and tested the effect of chemical inhibition on virion uptake where SLC3A2 was identified as a novel target (89). A role for SLC3A2 had been already described for hepatitis C virus infection (90). SLC6A15 is a neutral amino acid transporter, and it was recently connected to SARS-CoV-2 infection via photocatalytic proximity labeling of the Spike interactome and listed as a candidate auxiliary host entry factor (91). Interestingly, SLC6A15 was grouped with SLC6A19 and SLC6A20 (that are interacting with ACE2) in a recent review of SLCs (45), and it has been linked to the hippocampus and depression (92). SLC7A1 is a transporter for specific amino acids and captured in VThunter as a receptor for bovine leukemia virus (93). SLC20A1 is a sodium-phosphate symporter and featured in VThunter, whereby it serves as receptor for gibbon ape leukemia virus (94). SLC20A2 is an inorganic phosphate transporter, listed in VThunter, emerging from the DAVID analysis and listed as Gibbon Ape Leukemia Retrovirus Receptor 2 (MIM 158378). SLC29A3 is a nucleoside transporter (also known as ENT3) that was experimentally found to be required for SARS-CoV-2 viral genome release (95). SLC38A9 is involved in several transport functions, including amino acids, and it was very recently described to regulate SARS-CoV-2 viral entry (84), whereby the cleavage product S1 of Spike present in the endolysosome lumen may interact with SLC38A9, leading to increased escape of the virus from endolysosomes and enhanced viral entry. Two additional genes from the broader set of SLC-H were also of interest. SLC10A1 (NTPC) is a specific receptor for hepatitis B or D viruses (96). SLC52A2 was captured by DAVID analysis, and it was described as receptor for the Porcine endogenous retrovirus A (97). Extended literature searches for SLC-H-E and viruses in general detected additional links to infection mechanisms for other viruses as shown in Supplementary material 5, illustrating that a variety of substrates can be involved. In summary, evidence from several sources indicates roles of SLCs expressed in brain endothelial cells (as defined by SLC-H-E) in viral receptor functions, viral entry mechanisms, and other major roles in the viral cycle, and several SLCs with viral links are amino acid transporters.

### ABC transporters expressed in brain endothelial cells interact with SARS-CoV-2 proteins

Analysis of the VH-PPI for the term “ABC” resulted in *n* = 169 entries comprised of *n* = 23 unique ABC genes/proteins (Table 5). Of these *n* = 17 were expressed in brain endothelial cells based on the transcriptomics data with a cut-off at 10 TPM (bold in Table 5). All selected ABC genes were found listed in a database for brain expression (48). No significant difference was identified between the number of entries in VH-PPI for ABCs (at a cut-off level of 10 TPM) and SLC-H-E (t-test, *p* = 0.562). No difference was found between the two gene sets for expression levels in brain endothelial cells (*p* = 0.4).

**Table 5:** ABC transporters in virus-host protein–protein interactions.

**ABCA1**	**ABCB7**	ABCB11	**ABCC4**	**ABCD3**	**ABCF2**
**ABCA3**	ABCB8	**ABCC1**	**ABCC5**	**ABCD4**	**ABCF3**
**ABCB1**	ABCB9	ABCC2	**ABCC9**	**ABCE1**	**ABCG2**
ABCB6	**ABCB10**	ABCC3	**ABCC10**	**ABCF1**	

The table illustrates the ABC transporter proteins identified in VH-PPI (*n* = 23). ABC proteins with an expression level of *n* = 10 TPMs or higher in brain endothelial cells are indicated in bold (*n* = 17).

### Multiple host SLCs are shared between endothelial cells, astrocytes, and mural cells

**Figure 5:**
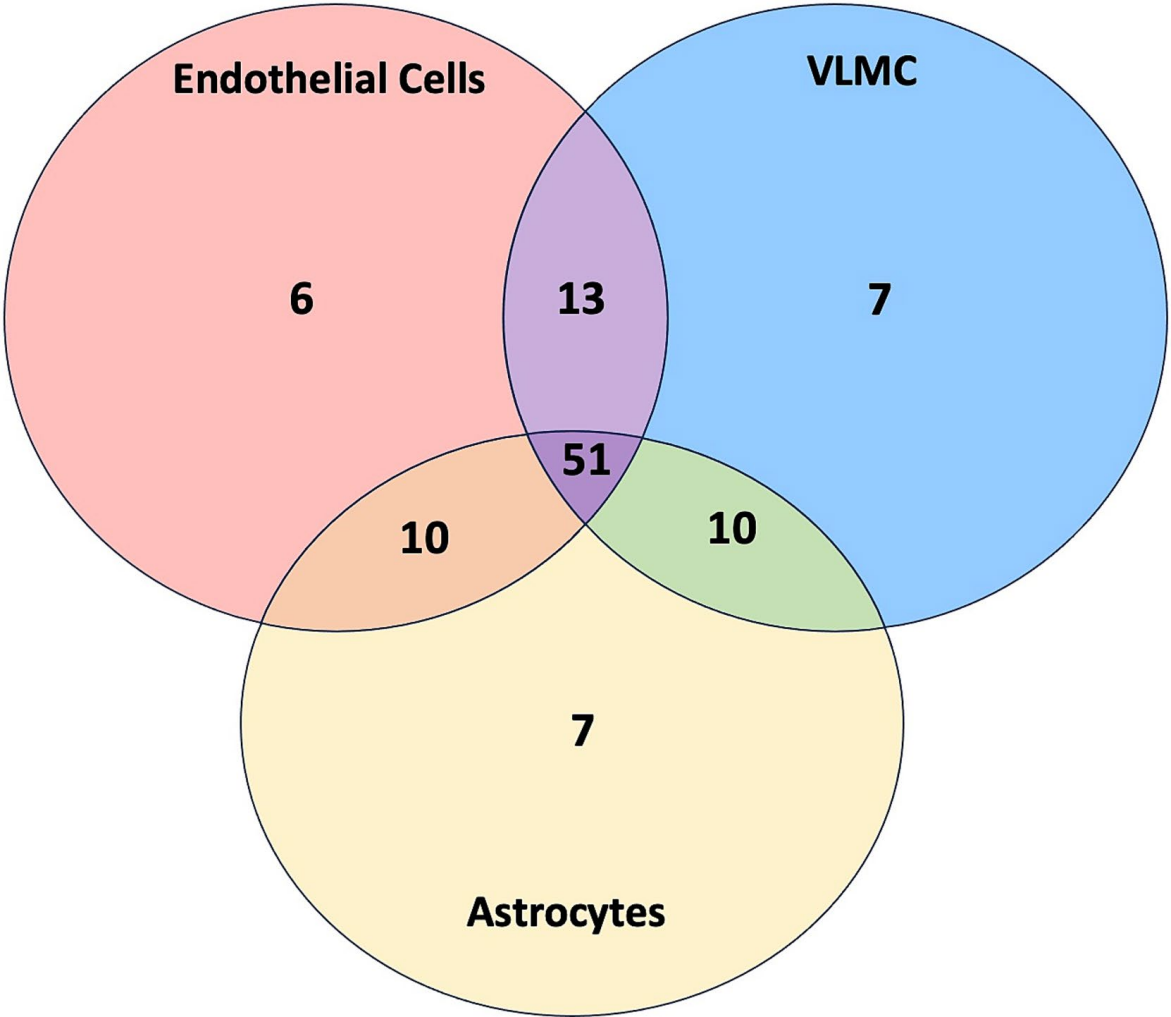
Venn diagram of SLCs in endothelial cells, VLMC, and astrocytes. The Venn diagram displays the number of SLCs belonging to each category, including endothelial cells (red), VLMC (blue), and astrocytes (yellow) as well as SLCs shared between multiple categories. Details are provided in Supplementary File 6.

The results of the combined analysis of SLCs interacting with viral proteins in endothelial cells, astrocytes, and mural cells (VLMC) are shown as a Venn diagram in Figure 5. The specific SLCs for each segment of the figure are listed in Supplementary material 6. The total numbers of selected SLCs were well-balanced among cell classes: endothelial cells (*n* = 80), astrocytes (*n* = 78), and mural cells (*n* = 81). The majority of SLCs were shared by all three cell types (*n* = 51), whereas much fewer SLCs were cell-specific: endothelial cells (*n* = 6), astrocytes (*n* = 7), and mural cells (*n* = 7). Next, the viral proteins (*n* = 26) were analyzed for interactions with SLCs whereby the number of interactions with SLCs was tabulated for each viral protein and cell type. Nine viral proteins (E, M, nsp4, nsp6, ORF3a, ORF3b, ORF7a, ORF7a, and S) had at least one interaction with SLCs for all three cell types. While the average number of interactions across all plotted viral proteins was highest for host SLCs from astrocytes (2.35), intermediate for mural cells (VLMC, 2.04) and lowest for endothelial cells (1.7), the differences were not significant (ANOVA, *F* = 0.37, *p* = 0.69). This analysis suggests that in addition to the central role of SLCs in endothelial cells (reached first by the virus during hematogenic spread), astrocytes and other vascular cells express SLCs relevant to viral infection with SARS-CoV-2. The shared SLCs could be defined as broad targets for interventions.

### Therapeutic strategies involving host SLCs

The annotation of SLCs interacting with SARS-CoV-2 viral proteins led to a number of specific transporters with key functions in endothelial cells. Such SLCs could be of interest for therapeutic interventions in COVID-19. SLC2A1 (GLUT1) is a key glucose transporter in brain endothelial cells, and dysfunction of this transporter due to a “block” by viral proteins could impact glucose levels in the brain. As mutations of SCL2A1 cause a clinical disorder, GLUT1 deficiency disorder (as listed under the analysis of OMIM), treatments may be already under development. Most prominently, *n* = 21 amino acid transporters were identified. Impairment of amino acid transport in brain endothelial cells could affect neuronal function, especially protein synthesis, which in turn is important for memory formation (98). Increasing amino acid delivery across the BBB may be tested as a strategy to overcome chronic, functional neurological changes after COVID-19 infection. Given the complex overlap of transported amino acids amongst different types of transporters and the number of transporters potentially affected, it is difficult to predict which amino acids would be most affected and should be targeted for supplementation. Theoretically, infusion with standard cocktails of amino acids used for parental nutrition could be tested in clinical trials. While this study mostly considered a decrease of transport functions by SLCs targeted by viral proteins, there is a theoretical possibility that viruses utilize host SLC functions to increase amino acid uptake for their own advantage. In this case, supplementation should be avoided. Nine SLCs serving as zinc transporters in brain endothelial cells were found to interact with viral proteins, and zinc has been discussed extensively in relation to COVID-19 (99, 100). Three SLCs serving as choline transporters were identified, and levels of choline in the corpus callosum were shown to be increased in patients affected by COVID-19 when measured with proton MR spectroscopy (101). It remains to be studied whether supplementation with choline would be indicated. The functional annotation tool DAVID returned “neurotransmitter transport” as a significant finding for the list of SLCs expressed in endothelial cells, and similarly Enrichr had “glutamatergic synapse” as a significant result. Transport of amino acids across the BBB may indirectly be connected to the neurotransmitter pools for glutamate and amino acid co-agonists at the NMDA receptor, which in turn is relevant to excitotoxic neuronal damage in viral infections (102, 103). SLC12A2 (NKCC1, Na-K-2Cl cotransporter 1) was detected as a host target shared by all three cell classes. NKCC1 regulates chloride levels important for the function of ionotropic GABA receptors, and NKCC1 is a drug target for neurodevelopmental disorders (104, 105). SLCs are major drug targets, and several compounds already exist to influence SLC functions, including in the brain (71, 105–107). Future studies should directly examine the function of SLCs after infection of brain endothelial cells to identify transport deficits that could be targeted. A caveat is that drugs blocking SLCs used for treatment of CNS disorders may work synergistically with the negative effects of viral proteins and should be critically evaluated. SLCs supporting viral entry and replication may become targets for drug therapy in COVID-19. ABC transporters play important roles in drug movements across the BBB, and abnormalities in ABC functions due to interactions with viral proteins could alter drug levels in the brain during infection. Pharmacogenomics studies have considered genetic variants of ABCs in relation to drug therapy of COVID-19 (108).

## Discussion

The rationale for this hypothesis-generating study is based on the critical role the brain vasculature and the BBB play in COVID-19 infection (17–33). Once endothelial cells become infected, viral proteins interacting with SLCs may impair transport of their substrates across the BBB, in particular of amino acids. The downstream effects of impaired transport will be decreased substrate delivery to neurons and glial cells, which impair protein synthesis due to reduced influx of amino acids. In conjunction with the systemic inflammatory response in COVID-19 (11–13), altered transport across the BBB could explain diffuse brain dysfunction, including cognitive deficits (14, 29). Perturbances of transmembrane transport may also affect cellular metabolism of brain endothelial cells themselves, possibly impair their viability, and add to the dysfunction at the BBB. In addition, inflammatory responses may cause abnormal interactions between endothelial cells, thrombocytes, and immune cells, leading to thrombosis followed by local ischemia (9, 39, 109). Disturbances of endothelial cells will be also sensed by perivascular astrocytic processes and microglial cells, contributing to the glial responses (neuroinflammation). Possible roles of selected SLCs as therapeutic targets have been addressed above under “Therapeutic strategies involving host SLCs.”

This study sought to identify a subset of SARS-CoV-2 viral proteins that interact with SLCs and then related these to known viral protein functions (64, 65). To this end, information for viral proteins was retrieved from a recent review of the innate immune evasion strategies of SARS-CoV-2 (65), and nine viral proteins interacting with host SLCs were categorized for key functions. This observation raises questions about how host SLCs are possibly involved in defense mechanisms through protein interactions, particularly interferon responses. Nsp6 emerged from the present analysis of host SLCs by matching all three categories for immune evasion, and interestingly, nsp6 mutations have been associated with the attenuation of infection seen in Omicron BA.1 variants (110). This study also examined ABC transporters expressed in brain endothelial cells as these proteins may serve as efflux transporters (48). Here, the concept was that inflammation may lead to a buildup of toxic substances within the brain parenchyma that need to be removed from the brain into the blood. Several ABCs were engaged in PPIs with viral proteins, perhaps suggesting decreased removal and intracerebral elevation of toxins. To further strengthen the argument for important functions of SLCs, monogenic disorders of the nervous system involving SLCs were also identified. Thereby, the idea was that mutations of SLCs may allow for predicting the sensitivity of SLCs to inhibitory protein interactions. Several SLCs related to disorders of the nervous system, in particular of the brain, were found. Given the involvement of white matter in COVID-19 (9), it is interesting that some genetic disorders of SLCs can affect the corpus callosum and the optic nerve. Some disorders involving SLCs can be classified as neurodevelopmental (111, 112), which may be of interest when studying the effects of SARS-CoV-2 on the developing brain (113).

The mechanisms of SARS-CoV-2 entry into cells involving the key receptor, ACE2, have been reviewed (114). More recently, several original reports have appeared for roles of SLCs in mechanisms of SARS-CoV-2 infections (44, 84, 87, 89, 91). Thereby, SLC1A5 stands out due to its involvement in other viral infections (44). In the present study, the DAVID analysis and literature evidence indicated that SLCs can be engaged in mechanisms of viral receptor functions, viral entry mechanisms, and other major roles in the viral cycle. This finding suggests a secondary role for SLCs serving in auxiliary functions in SARS-CoV-2 infection that may play out in brain endothelial cells. In fact, literature searches revealed that several SLCs are known to serve as cellular receptors or in closely associated functions for viruses other than coronavirus. Using the data for viral receptors in general from VThunter, it was found that SLCs are enriched among cellular receptors when considered on a genomic basis. It is intriguing that some of these SLCs serving in viral entry for other viruses are expressed in brain endothelial cells and are part of the interactome for SARS-CoV-2 proteins. The significance of this finding is presently unclear. Theoretically, prevention of viral interference could occur if SARS-CoV-2 viral proteins block SLCs used by other viruses as receptors which helps to avoid competing infections. SLCs may be broadly important for a number of viral infections, and some viruses (but not coronaviruses) may have specialized in recruiting specific SLCs as receptors. In the broader context, it is remarkable that ACE2, the key receptor for SARS-CoV-2, is known to interact with two amino acid transporters, SLC6A19 or SLC6A20, in enterocytes (45). These two proteins are not involved in direct interactions with SARS-CoV-2 viral proteins, and therefore, did not emerge from the present search in VH-PPI. Recent studies show that interaction with SLC6A20 may affect ACE2 receptor functions (115).

In the following, six limitations of this study need to be addressed.

1. This research combined data from studies in human cells at the protein level with mRNA data obtained in the mouse brain. The analysis was based on the annotations and listings in BioGRID, which use a unified symbol for the protein and gene based on SLC nomenclature; in addition, gene symbols were confirmed in the PANTHER database. Thereby, the assumption was made that SLCs are expressed in both mouse and human endothelial cells with a similar pattern and that they have closely related functions. The total number of SLCs was assumed to be n = 400 based on a review published in 2015 (34), which is close to the number of SLCs available in the mouse brain database (*n* = 382) published in 2018 (47). The list of SLCs is growing, in part due to new discoveries and the renaming of genes and proteins (116). The statistical analyses for enrichment of SLCs serving as viral receptors under VThunter depended on the total number used for the Chi-square tests. An estimate with a total number of SLCs of *n* = 450 showed that the enrichment remained significant (*p* = 0.0048) when referenced to 20 k genes, but became non-significant (*p* = 0.08) when referenced to 12 k proteins; this would not change the suggestion that SLCs play a role in viral receptor mechanisms. There is a theoretical possibility that a newly identified solute carrier has been given a protein name but has not yet been assigned a gene symbol beginning with “SLC,” and therefore was not detected. However, given the number of host SLCs with well-known functions in the BBB detected here, this would not change any conclusions.
2. The “SARS-CoV-2 and Coronavirus-Related Interactions” database within BioGRID contains a vast collection of interacting proteins that have been pooled from a large set of experiments using different types of human cell lines and different proteomic techniques. Some of these data are from work at the preprint stage. It is not clear to what extent some techniques have detected weak interactions in the respective assays, leading to an overestimation of the VH-PPI. No virus-host interactome data are available for cell lines closely resembling human brain endothelial cells (to the best of our knowledge). Since new studies with PPI information are regularly being added to the specialized BioGRID database, coverage was assumed to be complete. To test the validity of this assumption, individual studies of virus-host PPIs for SARS-CoV-2 were identified in PubMed abstract searches and successfully located in the BioGRID database. Since the present data analysis was closed by September 2024, newer studies for PPI data were also searched, and only one study was identified (117) in which a small set of SLCs was found, overlapping with the selection used here.
3. Theoretically, inactivation of host SLCs due to interaction with viral proteins may be compensated by the increased transcription of the same SLCs. Several studies have provided transcriptomics data of SARS-CoV-2 infected cells or post-mortem tissues whereby results for SLCs have been listed, e.g., for lungs (118). However, interpretation of such transcriptomics data is difficult, because a change of expression in SLCs, in particular down-regulation, could reflect generalized transcriptional failure and mRNA degradation caused by the acute viral take-over of cellular functions. On the other hand, it will be interesting to study expression levels of SLCs in the chronic state after COVID-19.
4. Only a few SLCs from VH-PPI data were also detected in CRISPR screens. The reason for this discrepancy could be that the read out from CRISPR screens is cell death upon viral infection, whereas no endpoint is used for the protein interaction data. A caveat is that the results from different CRISPR screens are not convergent and depend on the cell type used for screening (67). On the other hand, it will be interesting to test experimentally whether acute knock-down of several SLCs identified here could aggravate cellular deterioration after infection.
5. Several pathophysiological models have been based on the concept that SARS-CoV-2 infects brain endothelial cells and disturbs the BBB (17–33). Accordingly, the present study modeled the interaction of viral proteins with SLCs within infected brain endothelial cells, and the focus was on functional effects of transport across the BBB. It is conceivable that dysfunction of SLCs could also impair metabolism of endothelial cells themselves, which in turn could lead to an increased leakiness of the BBB, including viral entry into the brain parenchyma. Endothelial cells may be also indirectly involved by high levels of cytokines, inflammation and interaction with immune cells, which could impact metabolism and function of endothelial cells. Blood flow regulation could be impaired, leading to intermittent hypoperfusion and reduced oxygen delivery to neurons. To what degree SLCs in brain endothelial cells are affected by specific interactions with viral proteins and (or) by more general factors remains to be studied. It should also be noted that some experimental studies have challenged the concept that infection of brain endothelial cells by SARS-CoV-2 is a major factor (119, 120). Finally, the choroid plexus has been also established as an important site for SARS-CoV-2 infection of the brain (9, 13, 119); multiple SLCs are expressed in the choroid plexus which could be explored in the future using the strategies developed in the present study.
6. This analysis was focused on SLCs as one of the largest protein families with multiple important functions (34), but it should be recognized that many other protein families are involved in SARS-CoV-2 infection. The annotations and correlations presented for SLCs as such do not establish superior importance of these transmembrane transporters for SARS-CoV-2 infections. However, it is notable that SLCs are closely related to receptor functions for several viruses.

In conclusion, given the key role of the brain vasculature in neurological manifestations of COVID-19, the present hypothesis-generating work set out to analyze data for SLCs as important transporters at the BBB. By combining virus-host protein–protein interaction data and transcriptomics data for brain endothelial cells, a set of n = 80 host SLCs was prioritized for analysis. Amino acid transporters formed the largest group of SLCs involved. Information for brain disease associated with mutations of SLCs suggested importance for selected SLCs that may be impaired by viral protein interactions. Specific functions of viral proteins of SARS-CoV-2 interacting with SLCs were defined, including the interferon response. Impairment of SLCs in brain endothelial cells during the acute phase of COVID-19 may reduce substrate supply for neurons and glial cells. Cognitive changes in long-COVID could be theoretically caused by persistent dysfunction of SLCs at the BBB. An additional finding emerging from this study was that some SLCs play key roles in the infection mechanisms for SARS-CoV-2; this observation must be seen in the broader context that SLCs themselves can serve as receptors for other viruses.

## Data Availability

The original contributions presented in the study are included in the article/Supplementary material, further inquiries can be directed to the corresponding author.

## Supplementary material

Supplementary Material 1 The table displays the results of the PANTHER analysis which confirmed the Gene Name/Gene Symbol and identified the PANTHER Family/Subfamily and Protein Class for SLC-H-E and ABC proteins with an expression level of *n* = 10 TPMs or higher in brain endothelial cells.

Supplementary Material 2 The table displays the results of the Enrichr analysis which classified SLC-H-E into fifty-four functional categories and performed a Fisher Exact test.

Supplementary Material 3 The table extends Table 4 by providing the specific SLCs interacting with each viral protein selected based on a recent review (121). For the selection, refer to the text vand legend for Table 4.

Supplementary Material 4 The table displays the results of the OMIM™ Analysis which sought to identify SLCs associated with known monogenic disorders of the brain and their corresponding functions per NCBI Gene.

Supplementary Material 5 The table illustrates nine SLCs with connections to viruses other than SARS-CoV-2, including the specific virus involved, role during infection, substrates transported, literature source of the SLC-virus interaction, and associated PMID.

Supplementary Material 6 Table A illustrates the SLCs in endothelial cells, astrocytes, and VLMC as well as SLCs shared between the three categories. Additionally, Table B displays the number of interactions between SARS-CoV-2 viral proteins and SLCs in endothelial cells, astrocytes, and VLMC.
